# Pay-for-performance and low back pain with interaction of overwork: findings from the cross-sectional Korean working conditions survey

**DOI:** 10.3389/fpubh.2024.1364859

**Published:** 2024-05-20

**Authors:** Julia D. Hur, Jongin Lee

**Affiliations:** ^1^Department of Management and Organizations, New York University, New York, NY, United States; ^2^Department of Management and Organizations, New York University Shanghai, Shanghai, China; ^3^Department of Occupational and Environmental Medicine, Seoul St. Mary’s Hospital, College of Medicine, The Catholic University of Korea, Seoul, Republic of Korea

**Keywords:** low back pain, incentive reimbursement, psychological stress, occupational stress, working hour

## Abstract

**Background:**

Pay-for-performance (PFP) is a type of incentive system where employees receive monetary rewards for meeting predefined standards. While previous research has investigated the relationship between PFP and health outcomes, the focus has primarily been on mental health. Few studies have explored the impact of PFP on specific physical symptoms like pain.

**Methods:**

Data from the Korean Working Conditions Survey (KWCS) was analyzed, encompassing 20,815 subjects with information on PFP and low back pain (LBP). The associations between types of base pay (BP) and PFP with LBP were examined using multivariate logistic regression models, taking into account a directed acyclic graph (DAG). The interaction of overtime work was further explored using stratified logistic regression models and the relative excess risk for interaction.

**Results:**

The odds ratio (OR) for individuals receiving both BP and PFP was statistically significant at 1.19 (95% CI 1.04–1.35) compared to those with BP only. However, when the DAG approach was applied and necessary correction variables were adjusted, the statistical significance indicating a relationship between PFP and LBP vanished. In scenarios without PFP and with overtime work, the OR related to LBP was significant at 1.54 (95% CI 1.35–1.75). With the presence of PFP, the OR increased to 2.02 (95% CI 1.66–2.45).

**Conclusion:**

Pay-for-performance may influence not just psychological symptoms but also LBP in workers, particularly in conjunction with overtime work. The impact of management practices related to overtime work on health outcomes warrants further emphasis in research.

## 1 Introduction

Low back pain (LBP) is a common health problem prevalent in many people worldwide. According to the Global Burden of Disease Study, the age-standardized point prevalence of LBP decreased slightly from 8.2% in 1990 to 7.5% in 2017, but the number of affected individuals increased significantly from 377.5 million to 577.0 million (1). It is worth noting that LBP imposes a significant economic burden on society as a whole. In addition to direct medical expenses, indirect costs such as reduced work productivity are major contributors to the overall economic impact (2). Alongside the high prevalence rate, the cost of seeking medical care and losing time from work is also considerable (3). LBP also has a significant impact on the quality of life (4).

Certain diseases, such as lumbar disk herniation and spinal stenosis, can develop LBP. However, when disease-specific factors are excluded, it can be diagnosed as non-specific LBP, which accounts for 80–90% of LBP cases (5). The severity of pain in LBP can vary over time and can be attributed to several factors (6). Among them, biological, psychological, and social factors can act in combination to exacerbate LBP (7).

Occupational factors are also known to contribute to the occurrence or worsening of LBP, due to various musculoskeletal burdens. Reported factors include back bending and twisting, lifting and pulling heavy objects, and manual patient handling (8). There have been mixed findings on the impact of prolonged sitting on LBP in certain occupations: one meta-analysis reported a relationship between sedentary behavior and LBP (9), while some studies did not find such an association (10). Otherwise, female workers, long working hours, logistics and fishing workers, and psychosocial stress are known factors associated with LBP (11).

Various physical and psychological factors interact and contribute to musculoskeletal pain (12).

In a recent meta-analysis, a comprehensive examination of social determinants of health was conducted, including their association with LBP (13). Some studies cited in the meta-analysis included aspects of financial stability, while most studies focused on classifying socioeconomic status (SES) or income levels only.

Pay-for-performance (PFP) is a type of incentive system where employees receive monetary rewards for meeting predefined standards (14). PFP has been widely used in organizations across industries: in the United States, for example, 75% of companies have adopted some type of PFP system (15). The majority of previous research on PFP has been focused on the incentive system’s effect on motivation and performance (16, 17), as PFP was developed and applied to increase employee’s motivation and maximize their productivity. Recent work has started examining whether and how PFP affects employee’s quality of life and well-being in general (18, 19).

Relatedly, research has investigated a relationship between PFP and employee health outcomes. However, most of the previous work has focused on employee’s psychological symptoms, including stress, depression, and anxiety symptoms (20–23). For example, previous work with Danish firms found a 4–6 percent increase in the usage of antidepressant and antianxiety medication once a firm adopts PFP (22). Likewise, research with Korean working adults showed that PFP increased the risk of depressive and anxiety symptoms (23).

While recent research has seen a surge in investigating the relationship between PFP and mental health, there remains a dearth of studies examining the effect of PFP on specific physical symptoms, such as pain. Given how widely PFP has been adopted, it is important to understand its effect on physical health. Therefore, this study aimed to examine the relationship between PFP and LBP, a prevalent physical symptom that imposes a substantial disease burden.

## 2 Materials and methods

### 2.1 Study participants

The data for this study were obtained from the 6th Korean Working Conditions Survey (KWCS), conducted between 2020 and 2021 by Occupational Safety and Health Research Institute, Korea Occupational Safety and Health Agency, which encompasses Korean workers aged 15 years or older. The KWCS is on assessing the working conditions across the Korean workforce and examining their exposure to various work-related risk factors. The KWCS consists of similar questions licensed from the European Working Conditions Survey (EWCS) and is administered approximately every 3 years. The reliability and validity of the original dataset was introduced elsewhere (24). The response rate (RR3) for the 6th KWCS was 34.9%. Among the 50,538 respondents in the survey, this study specifically analyzed 33,063 individuals who were wage workers. Among the wage workers, 3,412 shift workers were excluded from the analysis, as their work schedule could have a significant effect on their working hours and other covariates. In the analysis of the wage workers, respondents who did not respond to questions regarding both PFP and LBP were excluded from the survey. Additionally, respondents who provided incomplete or invalid information regarding their monthly income were also excluded to adjust the effect of income level. As a result, a total of 20,815 subjects were included for this study (Supplementary Figure S1).

### 2.2 Covariates

The KWCS data was collected by conducting household visits, surveying the household members, and collecting information on their working conditions. Regarding the workplace information, the KWCS investigators subjectively recorded the name of the company and the tasks carried out by each household member. These tasks were then categorized into major occupation groups according to the International Standard Classification of Occupation (ISCO) with the help of experienced classifiers who had previously worked on the KWCS (Korean Working Conditions Survey). For the current study, the ISCO categories were consolidated into three groups by similarity suggested by Choi et al. (25): (1) Group 1–4 consisted of managers, professionals, technicians, and clinical support workers. (2) Group 5–6 included service, sales, agricultural, forest, and food workers. (3) Group 7–9 encompassed crafts, trades, machinery, and related occupations.

The current study investigated several factors related to work content, including: (1) High speed. (2) Tight deadlines. (3) Tiring or painful positions (excluding standing or sitting). (4) Lifting or moving people. (5) Carrying or moving heavy loads. (6) Prolonged standing. (7) Prolonged sitting. (8) Repetitive hand or arm movements. Each item was assessed based on the amount of time it took during the entire working hours. If a specific item accounted for more than half of the total working hours, it was considered as present or applicable for that individual. The study also included respondents’ job stress, which was evaluated based on the question “Do you experience stress in your work?,” with participants responding either “yes” or “no.”

Regarding working hours, the legal standard set by Korea’s Labor Standards Act is 40 h per week, with overtime work allowed up to 52 h. However, in some industries, working hours exceeding 52 h are either legally permitted or conducted illegally. Therefore, the study classified the respondents’ weekly working hours into three categories: less than 40 h, 40–52 h, and more than 52 h. The study gathered information on overtime work by analyzing the survey question: “Normally, how many times a month do you work?” If respondents indicated working at night, on Sundays, on Saturdays, or exceeding 10 h a day, it was classified as overtime work.

### 2.3 Pay-for-performance and low back pain

The KWCS included questions about the income of wage workers, specifically asking about the components of compensation from their main job. The survey investigated the following 10 detailed items: (1) Base salary/wage. (2) Piece rate. (3) Extra payments for additional hours of work/overtime. (4) Extra payments compensating for bad or dangerous working conditions. (5) Extra payments compensating for Sunday work. (6) Individual performance incentives. (7) Company-level performance incentives. (8) Regular bonus. (9) Individual commissions and tips. (10) Other non-monetary compensation (e.g., medical services, access to shops, etc.). We defined “Base salary/wage” as base pay (BP). We defined any of the 2, 6, 9 categories as pay-for-performance (PFP), following previous work defining PFP as the degree to which employees’ own, individual performance influences their pay. Table 1 describes the distribution of BP and PFP among the study respondents. Out of the total respondents, 15,792 (75.9%) received only BP, 4,447 received both BP and PFP, and 576 received wages with PFP only, without any BP.

**Table 1:** Number and proportion of type of income of survey participants.

Income type		BP only		BP + PFP		PFP only	
		(*N* = 15,792)		(*N* = 4,447)		(*N* = 576)	
Basic fixed salary/wage	No	–	(0.0%)	–	(0.0%)	576	(100.0%)
	Yes	15,792	(100.0%)	4,447	(100.0%)	–	(0.0%)
Piece rate	No	15,792	(100.0%)	2,067	(46.5%)	129	(22.4%)
	Yes	–	(0.0%)	2,380	(53.5%)	447	(77.6%)
Performance payments^*^	No	15,792	(100.0%)	1,306	(29.4%)	386	(67.0%)
	Yes	–	(0.0%)	3,141	(70.6%)	190	(33.0%)
Commission, Tips	No	15,792	(100.0%)	3,785	(85.1%)	537	(93.2%)
	Yes	–	(0.0%)	662	(14.9%)	39	(6.8%)
Income quartile	1st quartile (0–1.7 MKRW)	4,212	(26.7%)	276	(6.2%)	301	(52.3%)
	2nd quartile (1.7–2.4 MKRW)	4,381	(27.7%)	828	(18.6%)	98	(17.0%)
	3rd quartile (2.4–3.0 MKRW)	3,957	(25.1%)	1,317	(29.6%)	105	(18.2%)
	4th quartile (3.0–25.0 MKRW)	3,242	(20.5%)	2,026	(45.6%)	72	(12.5%)

^*^Individual or team based performance payments.

To define cases of LBP, individuals who answered “yes on backache” to the question “Over the last 12 months, did you have any of the following health problems?” were classified as having LBP. Responses such as “do not know,” “no opinion,” and “refused” were excluded from the analysis.

### 2.4 Statistical analysis

Chi-square tests were applied to explore the simple association between variables that could potentially be associated with LBP. These variables included gender, age, occupational group, working conditions, working hours, overtime work, and income type. To investigate the association between PFP and LBP, logistic regression analysis was conducted. Stepwise regression models, gradually adding variables such as PFP, gender, age, occupational group, working conditions, occupational stress, working hours, and overtime work, were applied. After that, to accurately calculate the total effect, a directed acyclic graph (DAG) was utilized in the regression analysis. This approach helps prevent errors such as over-adjustment and collider effects in the model correction (26). A multivariate logistic regression analysis model was then established to identify variables that were significantly related to PFP. These steps allowed us to take the specific relationships between PFP and the other variables into consideration. Because the inclusion criteria of study subject covered only complete cases of KWCS, the strategy for missing data was not considered.

In order to investigate the potential interaction between variables related to PFP and LBP, odds ratios were calculated using stratified logistic regression models. The variables considered for stratification were gender, age, overtime work, long working hours, income, fast work (defined as high speed or tight deadline), and job stress. This study is concerned with the interaction of different variables that affect LBP. To quantify the interaction, the Relative Excess Risk due to Interaction (RERI) and its confidence interval were calculated. In epidemiological studies, the RERI is a quantified indicator of how much more risk occurs when two exposure variables are present simultaneously than when they are present individually. For the calculation of the RERI, each variable was dichotomously divided. Among the six variables mentioned, long working hours were classified as more than 40 or 52 h per week, and fast work was defined as having either the high speed or tight deadline in the work. Age and income were divided into two groups based on their median values of 45 years old and 240 million Korean Won (MKRW). For sensitivity analysis, we ran the analysis by changing the definition of extremely long working hours to 55 h per week instead of 52 h per week.

The confidence level for all statistical analyses in this study was set at a *p*-value threshold of 0.05 and a confidence interval of 95%. The statistical analysis was performed using R version 4.3.1. (Vienna, Austria). The KWCS employed the clustered extraction method to obtain a representative sample of all Korean workers. To account for the survey’s design and sampling methodology, appropriate weights were applied to adjust for the clustering and ensure that the results accurately represent the entire population of Korean workers.

## 3 Results

The total number of subjects was 20,815, among which 5,412 individuals (26.0%) reported having LBP. The prevalence of LBP was higher in women (32.1%) compared to men (24.0%), and tended to increase with age, ranging from 11.9% in individuals under the age of 30 to 45.9% in those over the age of 60. Among occupational groups, the prevalence of LBP was highest among workers in the 7–9 category, which includes crafts, trades, machine operators, assemblers, and elementary occupations, often referred to as blue-collar workers. Regarding working conditions, most of the factors, including high speed, tight deadlines, tiring or painful positions (excluding standing or sitting), lifting or moving people, carrying or moving heavy loads, and inadequate movement directions, were associated with a higher prevalence of LBP. Job stress was also associated with a higher prevalence of LBP, but the association was not statistically significant. An increase in working hours and overtime work were both correlated with higher prevalence of LBP. Regarding payment systems, individuals who relied solely on PFP had the highest prevalence of LBP at 35.6%, whereas those receiving only BP and those receiving both BP and PFP had lower prevalence at 26.6 and 22.6%, respectively. Additionally, lower income levels were associated with a higher prevalence of LBP (Table 2).

**Table 2:** Basal characteristics associated with low back pain in KWCS participants.

	Low back pain				
	No		Yes		*p-*value
Total	15,403	(74.0%)	5,412	(26.0%)	
Gender					<0.001
Men	11,904	(76.0%)	3,760	(24.0%)	
Women	3,499	(67.9%)	1,652	(32.1%)	
Age					<0.001
<30	2,380	(88.1%)	323	(11.9%)	
30–40	3,832	(82.5%)	815	(17.5%)	
40–50	3,924	(76.8%)	1,184	(23.2%)	
50–60	3,330	(69.7%)	1,449	(30.3%)	
60 or above	1,937	(54.1%)	1,641	(45.9%)	
Occupational group					<0.001
1–4	9,572	(78.7%)	2,586	(21.3%)	
5–6	1,826	(81.1%)	425	(18.9%)	
7–9	4,005	(62.5%)	2,401	(37.5%)	
Working conditions					
High speed: No	11,367	(76.2%)	3,548	(23.8%)	<0.001
High speed: Yes	4,036	(68.4%)	1,864	(31.6%)	
Tight deadlines: No	11,469	(76.0%)	3,625	(24.0%)	<0.001
Tight deadlines: Yes	3,934	(68.8%)	1,787	(31.2%)	
Tiring positions: No	12,707	(79.4%)	3,301	(20.6%)	<0.001
Tiring positions: Yes	2,696	(56.1%)	2,111	(43.9%)	
Lifting or moving people: No	15,025	(74.8%)	5,064	(25.2%)	<0.001
Lifting or moving people: Yes	378	(52.1%)	348	(47.9%)	
Carrying heavy loads: No	14,051	(76.2%)	4,381	(23.8%)	<0.001
Carrying heavy loads: Yes	1,352	(56.7%)	1,031	(43.3%)	
Prolonged standing: No	8,295	(78.6%)	2,255	(21.4%)	<0.001
Prolonged standing: Yes	7,108	(69.2%)	3,157	(30.8%)	
Prolonged sitting: No	6,535	(69.9%)	2,820	(30.1%)	<0.001
Prolonged sitting: Yes	8,868	(77.4%)	2,592	(22.6%)	
Repetitive hand/arm move: No	8,017	(79.7%)	2,037	(20.3%)	<0.001
Repetitive hand/arm move: Yes	7,386	(68.6%)	3,375	(31.4%)	
Occupational stress: No	3,762	(75.4%)	1,230	(24.6%)	0.163
Occupational stress: Yes	11,641	(73.6%)	4,182	(26.4%)	
Working hours					<0.001
≤40	11,307	(75.2%)	3,731	(24.8%)	
>40 and ≤52	3,195	(71.9%)	1,251	(28.1%)	
>52	901	(67.7%)	430	(32.3%)	
Overtime work					<0.001
No	10,365	(76.6%)	3,165	(23.4%)	
Yes	5,038	(69.2%)	2,247	(30.8%)	
Income type					<0.001
BP only	11,590	(73.4%)	4,202	(26.6%)	
BP + PFP	3,442	(77.4%)	1,005	(22.6%)	
PFP only	371	(64.4%)	205	(35.6%)	
Income quartile					<0.001
1st (0–1.7 MKRW)	3,045	(63.6%)	1,744	(36.4%)	
2nd (1.7–2.4 MKRW)	3,968	(74.8%)	1,339	(25.2%)	
3rd (2.4–3.0 MKRW)	4,196	(78.0%)	1,183	(22.0%)	
4th (3.0–25.0 MKRW)	4,194	(78.5%)	1,146	(21.5%)	

In the logistic regression model analyzing the association between payment systems and LBP, the odds ratio (OR) was lower at 0.89 [95% confidence interval (CI) 0.79–1.00] insignificantly when having both BP and PFP. However, when relying solely on PFP, the OR significantly increased to 1.48 (95% CI 1.15–1.91). This trend shifted when adjusting for gender, age, occupational group, and income level. In model 1, which considered these variables, the OR for individuals with both BP and PFP was high with statistically significance at 1.19 (95% CI 1.04–1.35) compared to BP only. This trend persisted even when accounting for other factors related to LBP, such as working conditions, job stress, working hours, and overtime work. However, when the DAG approach was applied, the necessary correction variables to identify the total effect were found to be gender, age, occupational group, working hours, and overtime work (Supplementary Figure S2). In the model that adjusted these unbiasing variables, the statistical significance suggesting a relationship between PFP and LBP disappeared (Table 3).

**Table 3:** Stepwise multivariate logistic regression models and a DAG based model associated with low back pain.

Variable	Crude		Model 1		Model 2		Model 3		DAG based model	
	Odds ratio	(95% CI)	Odds ratio	(95% CI)	Odds ratio	(95% CI)	Odds ratio	(95% CI)	Odds ratio	(95% CI)
**Income type**										
BP only	(Ref)		(Ref)		(Ref)		(Ref)		(Ref)	
BP + PFP	0.89	(0.79–1.00)	1.19	(1.04–1.35)	1.18	(1.04–1.35)	1.19	(1.04–1.35)	1.12	(0.99–1.27)
PFP only	1.48	(1.15–1.91)	1.22	(0.94–1.58)	1.02	(0.78–1.33)	0.94	(0.72–1.23)	1.19	(0.92–1.53)
Gender										
Men			(Ref)		(Ref)		(Ref)		(Ref)	
Women			1.32	(1.18–1.48)	1.28	(1.14–1.45)	1.29	(1.14–1.45)	1.4	(1.25–1.57)
Age										
<30			(Ref)		(Ref)		(Ref)		(Ref)	
30–40			1.70	(1.38–2.09)	1.79	(1.45–2.21)	1.90	(1.53–2.35)	1.67	(1.36–2.05)
40–50			2.37	(1.93–2.91)	2.45	(1.98–3.02)	2.60	(2.10–3.21)	2.32	(1.90–2.84)
50–60			2.77	(2.28–3.38)	2.87	(2.34–3.51)	3.07	(2.50–3.76)	2.81	(2.31–3.41)
60 or above			4.23	(3.46–5.18)	4.89	(3.97–6.01)	5.36	(4.35–6.60)	5	(4.08–6.13)
**Income quartile**										
4th (3.0–25.0 MKRW)			(Ref)							
3rd (2.4–3.0 MKRW)			1.05	(0.92–1.21)	0.98	(0.85–1.14)	0.96	(0.84–1.11)		
2nd (1.7–2.4 MKRW)			1.21	(1.05–1.40)	1.18	(1.02–1.37)	1.18	(1.02–1.36)		
1st (0–1.7 MKRW)			1.34	(1.16–1.56)	1.48	(1.27–1.72)	1.64	(1.40–1.91)		
Occupational group										
1–4			(Ref)		(Ref)		(Ref)		(Ref)	
5–6			0.77	(0.65–0.90)	0.76	(0.65–0.90)	0.67	(0.57–0.79)	0.67	(0.57–0.78)
7–9			1.65	(1.48–1.83)	1.22	(1.08–1.38)	1.16	(1.03–1.30)	1.55	(1.39–1.72)
Working conditions										
High speed					1.15	(1.00–1.33)	1.12	(0.97–1.29)		
Tight deadlines					1.17	(1.01–1.34)	1.18	(1.03–1.35)		
Tiring positions					1.96	(1.75–2.19)	1.89	(1.69–2.11)		
Lifting or moving people					1.62	(1.27–2.07)	1.73	(1.36–2.20)		
Carrying heavy loads					1.52	(1.31–1.76)	1.46	(1.26–1.69)		
Prolonged standing					0.91	(0.81–1.01)	0.87	(0.77–0.97)		
Prolonged sitting					0.95	(0.85–1.06)	0.98	(0.87–1.10)		
Repetitive hand/arm move					1.26	(1.13–1.41)	1.28	(1.15–1.43)		
Occupational stress					1.44	(1.27–1.63)	1.40	(1.24–1.59)		
Working hours										
≤40							(Ref)		(Ref)	
>40 and ≤52							1.19	(1.04–1.37)	1.12	(0.99–1.28)
>52							1.31	(1.07–1.60)	1.31	(1.07–1.59)
Overtime work							1.54	(1.36–1.75)	1.65	(1.46–1.86)

In the analysis of factors related to PFP, women were less likely to report PFP compared to men, but this difference was not statistically significant (OR = 0.94, 95% CI 0.82–1.08). Regarding age groups, all higher age groups showed a significant association with PFP when compared to those under the age of 30. However, individuals over the age of 60 had a lower likelihood of reporting PFP (OR = 0.64, 95% CI 0.51–0.79). When comparing occupational groups, individuals in the 5–6 group, which includes sales workers, were more likely to report PFP, while those in the 7–9 group, consisting of blue-collar workers, were less likely to have PFP. Among the variables related to working conditions, tasks involving high speed, tight deadlines, tiring positions, and sitting were more likely to be associated with PFP. On the other hand, tasks involving prolonged standing and repetitive hand/arm movements showed a lower likelihood of being related to PFP. There was no significant association with working hours exceeding 52 h, but individuals working between 40 and 52 h had a lower likelihood of reporting PFP (OR = 0.85, 95% CI 0.75–0.97). There was a significant relationship between overtime work and PFP (OR = 1.24, 95% CI = 1.09–1.41), indicating that individuals working overtime were more likely to have PFP. Similarly, there was a significant association with job stress (OR = 1.31, 95% CI = 1.15–1.50), indicating that individuals experiencing job stress were more likely to report PFP (Table 4).

**Table 4:** Multivariate logistic regression model according to characteristics associated with pay-for-performance.

Variable		Odds ratio	95% Confidence Interval
Gender	Men	(Ref)	
	Women	0.94	(0.82–1.08)
Age	<30	(Ref)	
	30–40	0.97	(0.81–1.16)
	40–50	0.85	(0.72–1.02)
	50–60	0.89	(0.75–1.06)
	60 or above	0.64	(0.51–0.79)
Occupational group	1–4	(Ref)	
	5–6	2.25	(1.93–2.62)
	7–9	0.89	(0.78–1.02)
Income quartile	1st (0–1.7 MKRW)	(Ref)	
	2nd (1.7–2.4 MKRW)	1.25	(1.03–1.52)
	3rd (2.4–3.0 MKRW)	1.85	(1.53–2.24)
	4th (3.0–25.0 MKRW)	3.66	(3.02–4.43)
Working conditions			
High speed	Yes vs. No	1.2	(1.03–1.39)
Tight deadlines	Yes vs. No	1.29	(1.11–1.48)
Tiring positions	Yes vs. No	1.15	(1.00–1.31)
Lifting or moving people	Yes vs. No	1.49	(1.15–1.93)
Carrying heavy loads	Yes vs. No	0.96	(0.81–1.13)
Prolonged standing	Yes vs. No	0.57	(0.51–0.64)
Prolonged sitting	Yes vs. No	1.03	(0.91–1.15)
Repetitive hand/arm move	Yes vs. No	0.76	(0.68–0.84)
Working hours	≤40	(Ref)	
	> 40 and ≤52	0.85	(0.75–0.97)
	>52	0.89	(0.71–1.10)
Working holidays, night, long	No	(Ref)	
	Yes	1.24	(1.09–1.41)
Job stress	No	(Ref)	
	Yes	1.31	(1.15–1.50)

In the stratified model, the OR did not significantly change when considering only PFP in the case of no overtime work and applying PFP, compared to the reference of without PFP nor overtime work (OR = 1.01, 95% CI 0.87–1.17). However, when there was no PFP in the case of overtime work, the OR related to LBP was statistically significant at 1.54 (95% CI 1.35–1.75), and in the case of PFP, the OR increased to 2.02 (95% CI 1.66–2.45). This suggests an interaction between PFP and overtime work, but the RERI was not statistically significant at 0.25 (95% CI –0.32-0.81). Similar tendencies were observed with other variables, but the RERI was not statistically significantly high (Table 5).

**Table 5:** Multivariate logistic regression model according to characteristics associated with pay-for-performance.

Interaction variable		PFP	Odds ratio	95% Confidence Interval
Age	≤45	No	(Ref)	
RERI = −0.45	>45	No	2.29	(2.05–2.55)
95%CI = (−1.03–0.12)	≤45	Yes	1.40	(1.18–1.65)
	>45	Yes	2.00	(1.71–2.35)
Sex	Male	No	(Ref)	
RERI = −0.10	Female	No	1.44	(1.27–1.63)
95%CI = (−0.95–0.75)	Male	Yes	1.15	(1.02–1.31)
	Female	Yes	1.44	(1.07–1.94)
Income	> = Median	No	(Ref)	
RERI = 0.09	<Median	No	0.74	(0.66–0.83)
95%CI = (−0.39–0.57)	> = Median	Yes	1.06	(0.86–1.32)
	<Median	Yes	0.92	(0.79–1.06)
Overtime work	No	No	(Ref)	
RERI = 0.25	Yes	No	1.54	(1.35–1.75)
95%CI = (−0.32–0.81)	No	Yes	1.01	(0.87–1.17)
	Yes	Yes	2.02	(1.66–2.45)
Long working hours	No	No	(Ref)	
RERI = 0.14	Yes	No	1.10	(0.96–1.26)
95%CI = (−0.27–0.54)	No	Yes	1.07	(0.92–1.24)
	Yes	Yes	1.40	(1.16–1.68)
Fast work	No	No	(Ref)	
RERI = −0.07	Yes	No	1.59	(1.41–1.78)
95%CI = (−0.62–0.48)	No	Yes	1.14	(0.99–1.31)
	Yes	Yes	1.64	(1.36–1.98)
Job stress	No	No	(Ref)	
RERI = 0.19	Yes	No	1.35	(1.18–1.54)
95%CI = (−0.31–0.69)	No	Yes	0.91	(0.69–1.20)
	Yes	Yes	1.54	(1.31–1.82)

## 4 Discussion

This study represents the first attempt to establish a relationship between PFP and LBP to our best knowledge. By adjusting variables linked to PFP and LBP and applying multiple models, this study discovered that PFP was linked to a high prevalence of LBP, particularly through factors such as overtime work and extended work hours.

Covariates considered in this study align with existing knowledge about LBP. Specifically, the consistently higher prevalence of LBP among women and the escalating occurrence of LBP with age is consistent with previous knowledge. The significant prevalence of LBP among blue-collar workers also resonates with conventional understanding. The factors regarding work conditions, such as stressful situations including high speed, tight deadlines, and diverse ergonomic positions, were previously recognized to contribute to LBP, and this study reinforces those findings. Both long working hours and overtime work were also significantly associated with LBP in this study.

Long working hours are associated with a range of adverse health effects. Among the most widely recognized is cerebrovascular disease, which has been the subject of various studies, including a comprehensive meta-analysis by Kivimäki et al. (27). Suicide has also been studied, with the Japanese term ‘Karojisatsu’ describing the link between overtime work and suicide risk (28). Subsequent large-scale studies in the United States and Korea have reinforced the strong correlation between long work hours and an elevated risk of suicide (29, 30). However, comprehensive research encompassing all occupations and working hours related to LBP has been lacking. The relationship between working hours and LBP has been unequivocal in studies involving certain occupations like nurses, doctors, and those involving prolonged sitting (11, 31, 32). The odds ratio of 1.19 (95% CI 0.92–1.53) reported in the DAG-based model of this study was even larger, 1.51 (95% CI 1.21–1.89) in the same model (data not shown), when the definition of extremely long working hours was changed. Although the main results are reported based on a 52-h workweek to account for the definition of working hours in Korean legislation, the results of this sensitivity analysis suggest that working hours play a large role in the effect of PFP on LBP.

This study confirms the existing risks associated with long working hours and overtime, while also delving into the factors contributing to these hazardous work patterns. PFP has been commonly employed as a strategy to enhance corporate productivity, but adverse effects such as increased injuries and decreased productivity have been reported based on the specific application (33, 34). In this study, PFP was not associated with long working hours in itself; rather, the application of PFP was less prevalent in the 40 to 52-h workweek group compared to the group working 40 h or less per week. However, concerning overtime work—such as weekends, late-night shifts, and workdays exceeding 10 h—PFP exhibited a clear correlation. The reason for the unexpected relationship with total working hours might stem from the unique characteristics of the Korean work culture. When comparing the working environment survey in Korea and European countries, it was found that Korean workers work more hours than EU workers, but their intensity was low (35).

In the series of logistic regression models, when analyzed in the stepwise method, the effect of PFP did not show much difference between models. In the crude model, the OR of LBP was high in workers subject to only PFP, but in the adjusted stepwise models, the OR was about 1.19 and significantly higher when BP and PFP were applied at the same time. This effect was diluted only after the correct correction explored through DAG, and the consistent appearance of this effect suggests that there was a collider effect between working conditions, occupational stress, income level, and other variables considered as correction variables. Each variable interacts in a complex manner. For example, service jobs have a high rate of PFP application, and the income level is often low in condition that PFP is high. Given these complex interactions, it is suggested that LBP associated with PFP acts through overtime work as a mediator. Since PFP and LBP were significant in the generalization model, and overtime work was related to both PFP and LBP, it can be concluded that overtime work acts as a mediator by Baron and Kenny’s methodology (36). Recently, the mediating effect can be elaborately analyzed according to the methodology proposed by Hayes (37), but the result variable of this study was presented as LBP, a binary variable, so the limitation of the study is that this methodology cannot be applied. Instead, this study showed RERI, which is a method of calculating an excess risk and is not a test to determine the presence or absence of interaction. Although the RERI of the overtime work was estimated as 0.25 but the 95% CI included 0, the change in OR suggests that there is a possible interaction.

In addition, cross-sectional design is a limitation of this study. Various ergonomic risk factors showed a positive correlation with LBP, but in the case of prolonged sitting, it showed an opposite correlation. As mentioned in the introduction, previous results have been mixed on the LBP effect of sitting position. In this study, it was analyzed that the prevalence of LBP was rather low in the case of prolonged sitting, which may be because people with LBP avoid sitting for a long time. However, since the questions constituting the variables assumed in this study were carried out independently, the risk of inverse association is not expected to be significant. The definition of LBP was based on a survey, and there was no clear classification of its extent and scope. In addition, if a subject has a disease that can cause LBP, the subject should be excluded from the analysis; however, information on this was not known. But as mentioned in the introduction, about 90% of all LBP is non-specific LBP not caused by a specific disease: so there might be little difference in overall tendency even if the specific disease conditions were excluded. It is also a limitation of this study that important variables such as smoking were not considered, because it was not included in the KWCS. The relatively low response rate of KWCS is also a limitation. However, this study focuses on the associations among PFP, working hours, and LBP, which are unlikely to be influenced by variations in subject cooperation. Considering the large sample size, the association shown in this study could be applied to the general Korean population. However, it is necessary to replicate this study with different ethnic groups beyond the Korean population.

This study showed the underlying mechanism beyond the association of health effects caused by long-time and overtime work and showed that the PFP can affect not only psychological symptoms but also physical symptoms of workers. Employers often revise their PFP systems for managerial reasons. While these changes may be related to individual and organizational performance, the results of this study suggest that changes in PFP may be associated with health problems in organizational members. Therefore, monitoring physical symptoms, including LBP, is recommended when a company’s pay system changes. Research on management factors related to overtime work and health outcomes caused by them has been lacking so far. Future studies should be actively conducted in the related fields.

## Data availability statement

Publicly available datasets were analyzed in this study. This data can be found at: https://oshri.kosha.or.kr/eoshri/resources/KWCSDownload.do.

## Ethics statement

The studies involving humans were approved by Institutional Review Board of Seoul St. Mary’s Hospital, the Catholic University of Korea (Approval number: KC23ZNSI0600). The studies were conducted in accordance with the local legislation and institutional requirements. The ethics committee/institutional review board waived the requirement of written informed consent for participation from the participants or the participants’ legal guardians/next of kin because the study used nationally collected open data from Korea Working Conditions Survey.

## Author contributions

JH: Conceptualization, Methodology, Supervision, Writing – review & editing. JL: Conceptualization, Data curation, Formal analysis, Writing – original draft.
