# Supplementary figures and images for: Pay-for-performance and low back pain with interaction of overwork: findings from the cross-sectional Korean working conditions survey
Source: Front Public Health. 2024 May 20;12:1364859. doi: 10.3389/fpubh.2024.1364859 (PMC11144914; doi:10.3389/fpubh.2024.1364859)

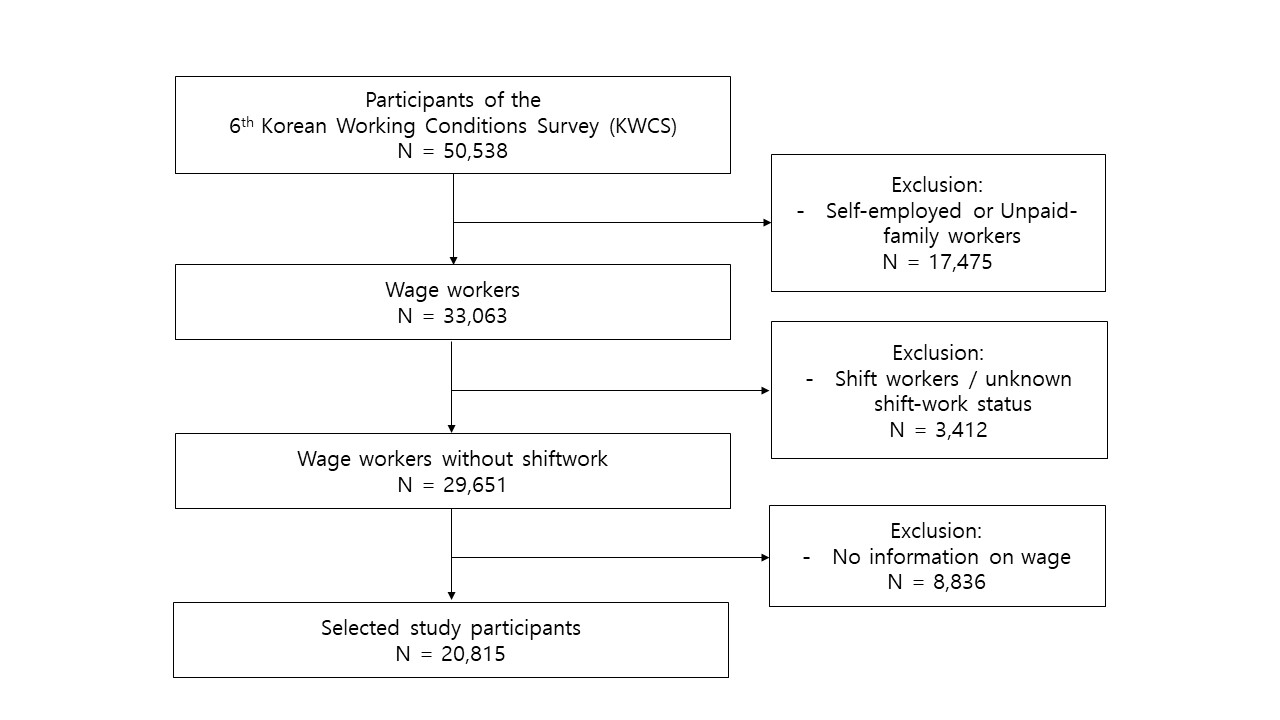

Supplement: Supplementary file 1 [file Image_1.JPEG]

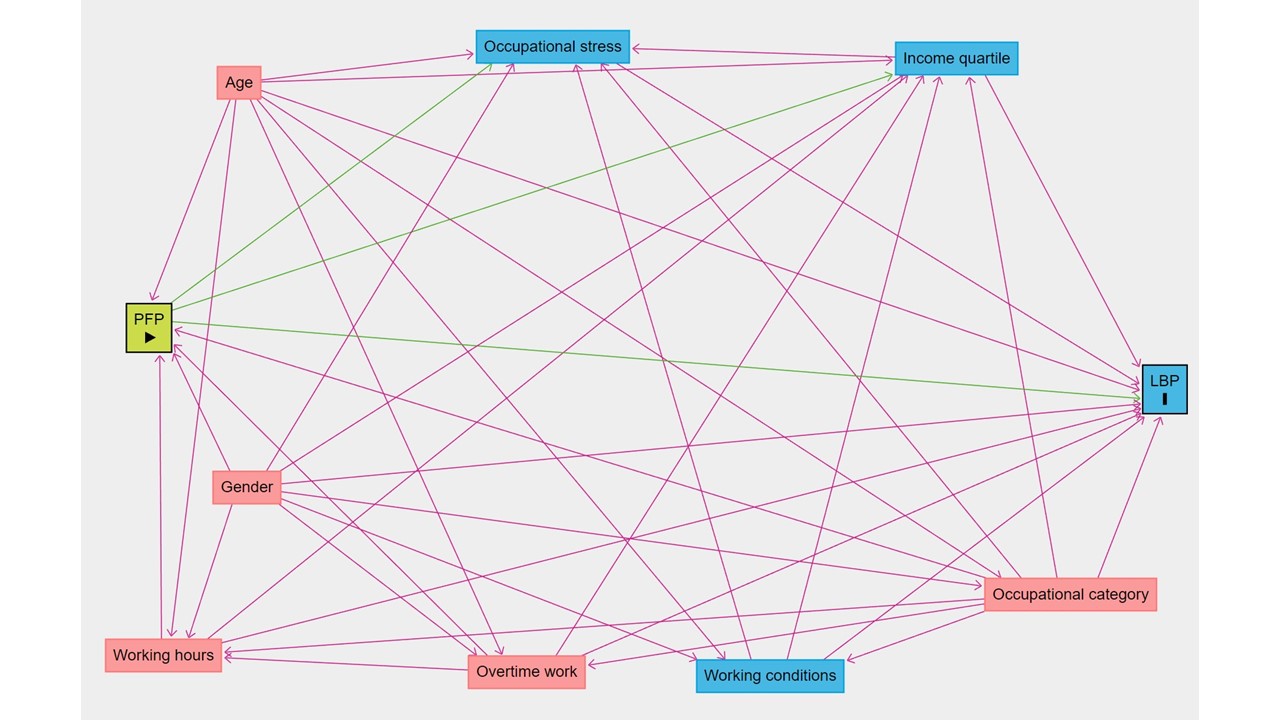

Supplement: Supplementary file 2 [file Image_2.JPEG]
